# Cytosolic acetyl-CoA: a metabolic regulator of mitophagy

**DOI:** 10.1093/lifemeta/loag002

**Published:** 2026-01-23

**Authors:** Jianhua Xiong

**Affiliations:** Department of Urology, Emory University School of Medicine, Atlanta, GA 30322, United States; Winship Cancer Institute of Emory University, Atlanta, GA 30322, United States

**Acetyl-coenzyme A (acetyl-CoA) is a central metabolite that ­underpins energy production, biosynthesis, and protein acetylation. A recent study by Zhang *et al.* published in *Nature* reveals a striking non-canonical role for cytosolic acetyl-CoA as a direct small-molecule ligand for the nucleotide-binding ­oligomerization domain (NOD)-like receptor NLRX1, stabilizing its autoinhibited state and thereby tuning receptor-mediated mitophagy [1].** 

**Figure 1:**
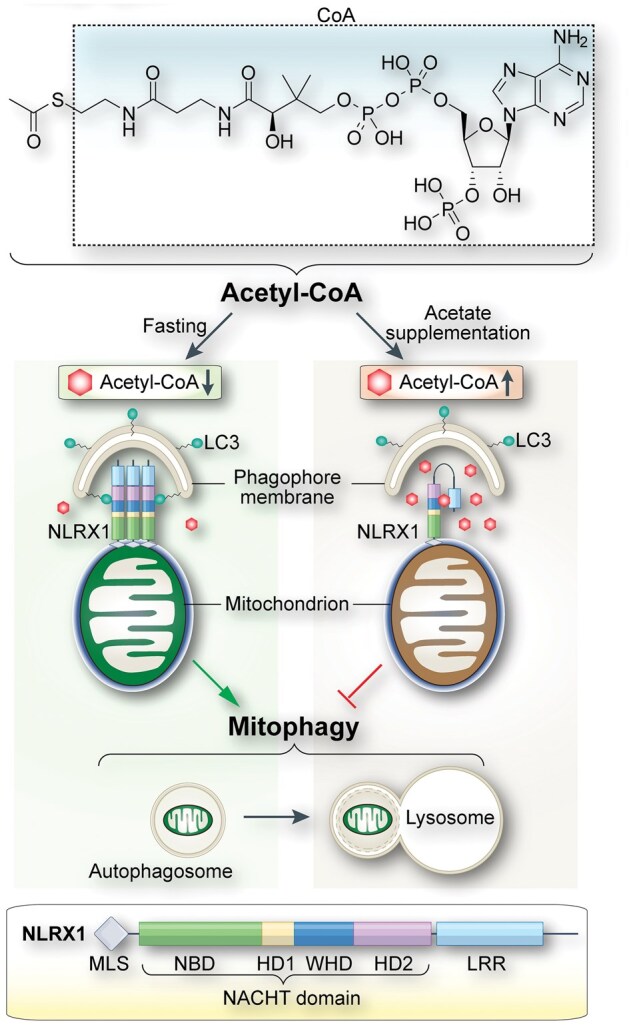
Cytosolic acetyl-CoA regulates NLRX1-mediated mitophagy. Top: acetyl-CoA, a membrane-impermeant metabolite that links nutrient status to mitochondrial quality control and consists of an acetyl group (CH_3_CO) linked via a thioester bond to CoA. Middle: the model of cytosolic acetyl-CoA linking cellular metabolic state to mitophagy. High cytosolic acetyl-CoA, maintained by nutrient sufficiency or acetate supplementation, binds NLRX1 LRRs, stabilizing an autoinhibited conformation. Low cytosolic acetyl-CoA, induced by fasting or inhibition of ACLY, ACSS2, or SLC25A1, releases NLRX1 autoinhibition, allowing LC3 recruitment, autophagosome assembly, and selective mitophagy. This pathway is cytosol-specific and AMPK−mTORC1-independent, and requires core autophagy machinery. Bottom: NLRX1 domains including MLS, NACHT (NBD, HD1/HD2, and WHD), and LRR with the acetyl-CoA-binding pocket. Abbreviations: HD1, helical domain 1; HD2, helical domain 2; LC3, microtubule-associated protein 1 light chain 3; LRR, leucine-rich repeat; MLS, mitochondrial localization signal; NBD, nucleotide-binding domain; NLRX1, NOD-like receptor X1; WHD, winged-helix domain.

Metabolism, the network of chemical reactions sustaining life, ­remains at the heart of chemical biology, as exemplified by more than 20 Nobel Prizes in Chemistry and Physiology or Medicine [2]. At the crossroads of metabolic networks lies acetyl-coenzyme A (acetyl-CoA), a membrane-impermeant molecule composed of an acetyl group linked to CoA via a high-energy thioester bond (Fig. 1). Classically, acetyl-CoA fuels the tricarboxylic acid cycle, supports fatty acid and lipid synthesis, and provides the sole acetyl donor for histone and non-histone protein acetylation, thereby coupling nutrient availability to gene regulation and protein function [3]. The study by Zhang *et al.* extends this paradigm by demonstrating that acetyl-CoA itself, independent of downstream acetylation events, can act as a signaling metabolite that directly modulates protein activity [1].

By experimentally lowering cytosolic acetyl-CoA through short-term fasting or pharmacologic and genetic inhibition of ATP citrate lyase (ACLY), the mitochondrial citrate transporter SLC25A1, or acyl-CoA synthetase ACSS2, Zhang *et al.* observed robust induction of mitophagy across multiple cell types [1]. Importantly, acetate supplementation restored cytosolic acetyl-CoA and reversed mitophagy, establishing acetyl-CoA as the operative signal rather than secondary effects of energy stress. This response occurred independently of canonical nutrient-sensing pathways involving AMP-activated protein kinase (AMPK) and mechanistic target of rapamycin complex 1 (mTORC1), distinguishing it from general autophagy induced by starvation. Moreover, the effect was highly selective: in nucleotide-binding oligomerization domain (NOD)-like receptor X1 (NLRX1)-deficient cells, mitophagy was markedly impaired while bulk autophagy remained largely intact [1]. These findings define a metabolic checkpoint in which cytosolic acetyl-CoA levels directly govern mitochondrial turnover.

Central to this mechanism is the identification of NLRX1 as the molecular sensor that links acetyl-CoA to mitophagy. NLRX1 is a member of the mammalian NOD-like receptor family, cha­racterized by a central NACHT ATPase domain and C-terminal leucine-rich repeats (LRRs) [4]. Rather than emphasizing its broader immunological history, Zhang *et al.* positioned NLRX1 functionally as a mitophagy receptor whose activity was gated by metabolic state. They demonstrated that NLRX1 directly interacted with microtubule-associated protein 1 light chain 3 (LC3) to recruit autophagosomes to the mitochondria and that this interaction was suppressed when cytosolic acetyl-CoA was abundant. Biochemical and structural analyses revealed that acetyl-CoA bound directly to a conserved pocket within the LRR domain of NLRX1, stabilizing an autoinhibited conformation through intramolecular coupling to the NACHT domain (Fig. 1). When cytosolic acetyl-CoA levels declined, this inhibition was relieved, enabling NLRX1−LC3 engagement and initiation of mitophagy [1].

Mechanistically, *in vitro* pull-down assays combined with in silico docking identified four conserved residues, Glu729, Lys754, Gln758, and Arg958, that were essential for acetyl-CoA binding. Mutation of these residues abolished ligand binding, confirming a direct molecular interaction. The isolated LRR domain bound acetyl-CoA with micromolar affinity (Kd ∼6.6 μmol/L), well within physiological cytosolic concentrations, firmly establishing NLRX1 as a bona fide small-molecule sensor. Importantly, related CoA metabolites such as malonyl-CoA and succinyl-CoA failed to bind NLRX1, underscoring the specificity of this interaction [1].

The authors further demonstrated that acetyl-CoA-­mediated mitophagy exhibited striking cellular specificity [1]. Under mild starvation or inhibition of cytosolic acetyl-CoA production, mitophagy-responsive cells, including HeLa, A549, and MCF7, displayed a selective reduction in cytosolic, but not mitochondrial, acetyl-CoA (Fig. 1). This decrease was correlated with LC3 recruitment to the mitochondria, mitochondrial DNA depletion, and reduced mitochondrial protein abundance. In contrast, U-2 OS osteosarcoma cells maintained higher basal cytosolic acetyl-CoA and failed to initiate mitophagy under identical conditions, highlighting how intrinsic metabolic states dictate responsiveness. Compartmental specificity was further reinforced by pH-dependent binding: the more alkaline mitochondrial matrix (pH ∼8.0) markedly reduced NLRX1−acetyl-CoA interaction, indicating that NLRX1 primarily senses cytosolic acetyl-CoA pools.

Notably, depletion of cytosolic acetyl-CoA did not compromise core mitochondrial functions, including membrane potential, ATP production, reactive oxygen species generation, protein import, or oxidative phosphorylation. Mitophagy induction nevertheless required intact autophagic machinery (Fig. 1), as knockdown of FIP200 or ATG7 blocked the process [1, 5]. Together, these findings demonstrate that NLRX1 responds to a precise metabolic cue rather than generalized mitochondrial dysfunction, allowing cells to fine-tune mitochondrial quality control in response to nutrient flux.

The physiological relevance of this pathway is supported by *in vivo* studies [1]. Short-term fasting or pharmacologic inhibition of ACLY in mice selectively reduced cytosolic acetyl-CoA in skeletal muscle and brain, triggering mitophagy in these tissues while sparing the liver. Restoration of cytosolic acetyl-CoA through ­acetate supplementation reversed mitophagy without altering ­mitochondrial biogenesis, reinforcing the idea that acetyl-CoA functions as a signaling metabolite rather than a simple biosynthetic intermediate.

Beyond physiology, the acetyl-CoA−NLRX1 axis has important therapeutic implications. Zhang *et al.* showed that treatment with Kirsten rat sarcoma viral oncogene homolog (KRAS) inhibitors suppressed the ACLY−acetyl-CoA pathway, thereby activating NLRX1-dependent mitophagy [1]. Given that KRAS mutations drive a large fraction of human cancers and that acquired resistance limits the efficacy of KRAS-targeted therapies, this finding identifies metabolically regulated mitophagy as a mechanism of drug tolerance. Combining KRAS inhibition with pharmacologic suppression of mitophagy synergistically impairs cancer cell growth, highlighting the ACLY−acetyl-CoA−NLRX1 pathway as a potential target for therapeutic sensitization [6].

More broadly, this study established cytosolic acetyl-CoA as a bona fide signaling metabolite that linked cellular metabolic state directly to mitochondrial quality control. It exemplified a fundamental biological principle: small-molecule metabolites could act as direct ligands to modulate protein conformation and activity, bridging metabolism, intracellular signaling, and organelle homeostasis. By connecting a core metabolic intermediate to selective mitophagy, this work reveals a previously underappreciated layer of metabolic regulation with broad implications for health and disease. These principles likely extend beyond cancer cells to diverse contexts, including immune regulation, vascular function, tissue aging, neurodegeneration, and metabolic disorders [7–10].

Looking ahead, several important questions emerge. Do ­other central metabolites, such as succinyl-CoA, malonyl-CoA, or NAD^+^ derivatives, serve as direct ligands for NLRX1 or related receptors, creating a broader metabolic language to fine-tune mitophagy and organelle homeostasis? How do post-translational modifications, co-factors, and tissue-specific programs influence acetyl-CoA sensing and NLRX1 activity? Do these circuits vary with systemic metabolic states such as fasting, obesity, or caloric restriction, and do they contribute to aging or age-related diseases?

The translational potential is compelling. Small-molecule modu­lators, synthetic ligands, or targeted dietary interventions could precisely tune NLRX1-mediated mitophagy, offering strategies to improve mitochondrial quality control in cancer, metabolic disorders, or neurodegeneration. Coupling mitophagy modulation with existing therapies such as KRAS inhibitors may enhance efficacy and overcome resistance.

Ultimately, this study frames metabolites not only as fuels or substrates but as active regulators of organelle dynamics, stress adaptation, and tissue resilience. Understanding these metabolite-driven signaling networks will deepen our knowledge of cellular metabolism, inform interventions to modulate aging and disease, and inspire precision strategies to harness metabolic control for therapeutic benefit.
